# Longitudinal changes in DNA methylation in IDH-mutant glioma fuel disease progression through altered cell state differentiation

**DOI:** 10.1038/s41588-026-02642-7

**Published:** 2026-06-22

**Authors:** Masashi Nomura, Ramya Raviram, Joshua S. Schiffman, Lillian Bussema, Vivian Lu, Noelle Wheeler, John J. Y. Lee, Yilin Fan, Mian Hua Zheng, Florian Ruiz, Husain Danish, Sorcha Kellett, Labeeba Nusrat, Ronan Chaligne, Jason T. Huse, W. K. Alfred Yung, Shota Tanaka, Nobuhito Saito, Sunit Das, Catherine Potenski, Dan A. Landau, Mario L. Suvà

**Affiliations:** 1 Department of Pathology and Krantz Family Center for Cancer Research, Massachusetts General Hospital and Harvard Medical School, Boston, MA USA; 2 Broad Institute of Harvard and MIT, Cambridge, MA USA; 3 Department of Neurosurgery, Graduate School of Medicine, The University of Tokyo, Tokyo, Japan; 4 New York Genome Center, New York, NY USA; 5 Weill Cornell Medicine, New York, NY USA; 6 Division of Clinical Pathology, Geneva University Hospital, Geneva, Switzerland; 7 Division of Neurosurgery, St. Michael’s Hospital, University of Toronto, Toronto, Ontario Canada; 8 Computational and Systems Biology Program, Sloan Kettering Institute, Memorial Sloan Kettering Cancer Center, New York, NY USA; 9 Department of Pathology, The University of Texas MD Anderson Cancer Center, Houston, TX USA; 10 Department of Neuro-Oncology, The University of Texas MD Anderson Cancer Center, Houston, TX USA; 11 Department of Neurological Surgery, Graduate School of Medicine, Dentistry and Pharmaceutical Sciences, Okayama University, Okayama, Japan

**Keywords:** CNS cancer, Epigenetics

## Abstract

The progression of isocitrate dehydrogenase-mutant glioma (IDH-G) from slow-growing tumor to fatal disease is associated with transcriptional and DNA methylation changes that remain poorly understood. Here, we profiled a longitudinal cohort of 36 IDH-G samples from 19 patients by joint-capture multi-omic single-nucleus DNA methylation, single-nucleus RNA sequencing and bulk exome sequencing. We show that IDH-G progression is associated with an increase in malignant stem-like states, decreased differentiation and methylation loss, which marks tumors with worse clinical outcome. Methylation loss was uniformly observed across malignant cells within individual tumors, suggesting that it may underlie rather than result from the increase in stem-like states. Analysis of cell-state heritability and plasticity using high-resolution phylogenetic trees links DNA methylation loss to alterations in glioma cell-state encoding and heritability. Our study offers insights into how DNA methylation loss reshapes cellular transitions and how it may mark clinically more aggressive tumors across IDH-G subsets.

## Abstract (web summary)

The authors use single-cell multi-omics to interrogate longitudinal samples of isocitrate dehydrogenase mutant gliomas. Their analyses couple progression and hypomethylation with changes in differentiation topologies and increased emergence of stem-like states in aggressive disease.

## Main

Mutations in isocitrate dehydrogenase *IDH1* and *IDH2* define a subset of adult diffuse gliomas (IDH-G) with distinct clinical, morphological and molecular features^1–3^. *IDH1/2* hotspot mutations promote gliomagenesis by the over-accumulation of D-2-hydroxyglutarate (D-2HG), a competitive inhibitor of α-ketoglutarate-dependent dioxygenases, such as histone and DNA demethylases^4–6^. This leads to genome-wide hypermethylation that also affects CpG islands, known as the glioma CpG islands methylator phenotype (G-CIMP)^7,8^. It has been proposed that changes in DNA methylation promote tumor fitness by hindering cellular differentiation, silencing tumor suppressor loci such as *CDKN2A* or disrupting insulation of the *PDGFRA* oncogene locus, and that D-2HG may lead to non-cell-autonomous T cell dysfunction^5,6,9,10^.

IDH-Gs typically present as low-grade, slow-growing tumors, but invariably progress to high-grade^11^. This progression is associated with acquired genetic alterations, such as the acquisition of genome-wide copy number aberrations (CNA) and mutations affecting oncogenes and tumor suppressors (for example, *CDKN2A*)^12–16^. Additionally, IDH-G progression is associated with changes in malignant cell state proportions, with an increase in stem-like states (neural progenitor cell-like and oligodendrocytic precursor cell-like) and a decrease in differentiated states (astrocyte (AC)-like and oligodendrocyte (OC)-like)^17–20^. IDH-G progression is also associated with global changes in DNA methylation, and subsets of progressed tumors display loss of G-CIMP status (G-CIMP-low), a switch associated with shorter survival^21–25^. How a switch to G-CIMP-low leads to a more aggressive tumor phenotype is not understood. Equally unaddressed is how epigenetic or genetic variation quantifiably contributes to the evolution of cellular phenotypes over cell generations and disease progression.

Multimodal single-cell RNA sequencing (scRNA-seq) and DNA methylation studies have begun to address those questions and reveal the epigenetic encoding of IDH-G cell states, offering a more direct analysis of cell plasticity and heritability by incorporating ancestral information with phenotypic profiling. One study in particular^26^ highlights a mostly hierarchical tumor architecture for IDH-G, with differentiation of stem-like states towards the AC-like and OC-like lineages and only limited de-differentiation. Despite this progress, major gaps remain in understanding the molecular underpinnings of the diverse malignant cell states that drive IDH-G, their dynamics and evolution. Existing scRNA-seq^15,17,19^ or multimodal scRNA-seq and single-cell DNA methylation studies^26^ were constrained by a limited number of samples and cells, did not have matched longitudinal samples (and therefore could only indirectly interrogate tumor progression) and lacked in-depth genetic characterization of the tumors. As such, it remains unclear how epigenetic or genetic variation quantifiably contributes to the evolution of cellular phenotypes in IDH-G progression.

Here, we leverage recent advances in single-nucleus multi-omics technologies to profile a longitudinal cohort of 36 matched IDH-G tumor samples from 19 patients. We applied joint capture of full-length transcriptional (by Smart-seq2, SS2 (ref. ^27^)) and epigenetic information (by extended-representation bisulfite sequencing (XRBS^28^)). Compared to prior multi-omic single-cell technologies^26,29,30^, this approach provides higher coverage outside of CpG islands, which is critical to interrogate DNA methylation changes outside of promoter regions. Our analyses confirm changes in cell state frequency during IDH-G progression, with an increase of stem-like states and a decrease in differentiated malignant cells, and identify potential transcriptional and epigenetic cell state regulators, such as hypomethylation of PRC2 targets and increased expression of glioma stem-cell genes. Single-nucleus DNA (snDNA) methylation analysis supports a global reduction in progressed tumors but shows comparable DNA methylation levels between different malignant cell states within individual tumors. This observation suggests that decreased DNA methylation may underlie rather than result from a change in cell state composition. To address whether decreased G-CIMP status in progressed tumors affects cellular transitions, we apply a quantitative framework to directly measure cell state heritability based on transcriptional annotation of high-resolution phylogenetic trees. Our analysis suggests that G-CIMP-low tumors transition to decreased cellular differentiation and increased heritability of stem-like states, potentially leading to increased tumor fitness. Importantly, we show that progression to G-CIMP-low tumors occurs in both astrocytoma and oligodendroglioma and is associated with worse clinical outcome. This work integrates transcriptional cell states with their epigenetic encoding and transitions during glioma progression, offering insights into the functional impact of changes in DNA methylation on glioma evolution.

## Results

### Single-nucleus multimodal analysis of a longitudinal cohort of IDH-G

**Fig. 1:**
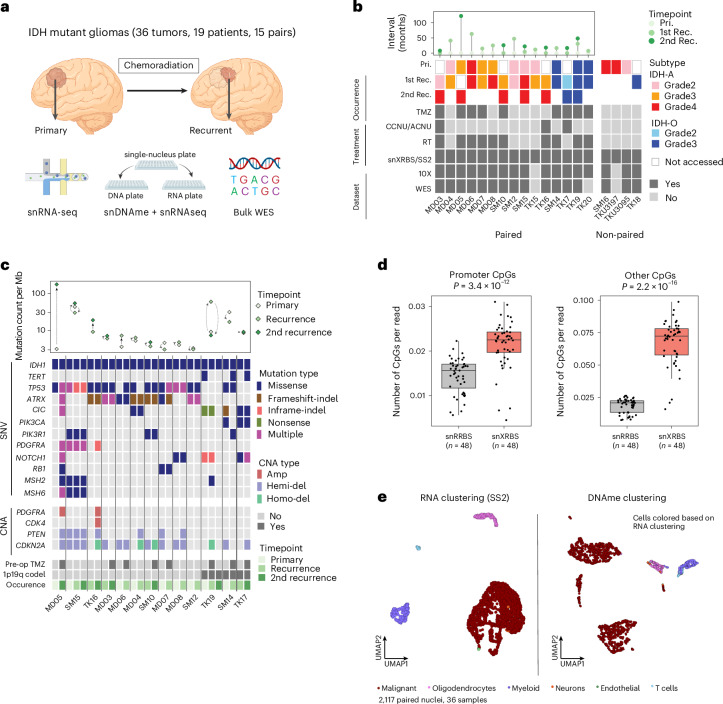
Dataset and study workflow. **a**, Scheme depicting the workflow of this study. snDNAme, single-nucleus DNA methylation sequencing. Created in BioRender; Nomura, M. https://BioRender.com/ju18r6e (2026). **b**, Clinical characteristics and dataset information of the cohort in this study. Pri., primary; Rec., recurrence; TMZ, temozolomide; RT, radiotherapy. **c**, Genomic information of the matched-pair samples analyzed by WES. The top panel shows the number of somatic mutations. Each diamond reflects a sample. The color reflects the timepoint. The middle panel shows whether known driver mutations and CNAs exist or not in the early timepoint and late timepoint. Color reflects the type of mutation and CNA. The bottom panel shows the clinical information. SNV, single nucleotide variant; indel, insertion and deletion; Homo-del, homozygous deletion; hemi-del, hemizygous deletion; amp, amplification; pre-op, pre-operative; codel, co-deletion. **d**, Captured promoter CpGs normalized by reads (left) and captured non-promoter CpGs normalized by reads (right). Each dot reflects a cell. Boxplots span from the first to third quartiles, with the median indicated by a horizontal line and whiskers extending to 1.5× the interquartile range (IQR). Statistical significance was estimated using a two-sided Mann–Whitney *U*-test. **e**, Clustering of snRNA-seq data based on variable genes (left) and DNA methylation based on 100 kb bins (right). Cell type labels were determined based on correlation with normal brain cell types and CNAs in malignant cells.

**Extended Data Fig. 1:**
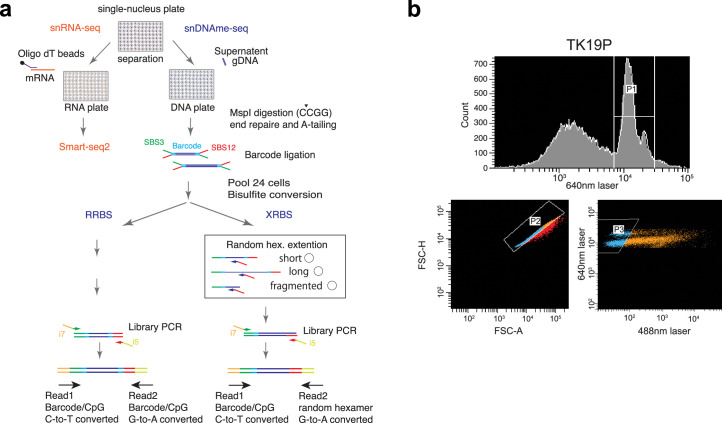
Workflow of single-nucleus joint transcriptome and DNA methylation sequencing. **a**, Schematic for the flow of single-nucleus joint transcriptome and DNA methylation sequencing, with illustration on the differences in workflow between RRBS and XRBS (this study). By adding a random hexamer extension step to the RRBS, we successfully amplified long and fragmented products as shown in the study by Shareef et al.^28^, resulting in higher coverage. **b**, Gating strategy for single nuclei sorting for joint transcriptome and DNA methylation sequencing protocol. First, ruby positive fraction (P1, top) was selected by 640 nm laser. Then, doublet discrimination was performed by strict forward scatter height (FSC-H) versus area (FSC-A) criteria (P2, bottom left). It was further gated by removing 480 nm laser positive fraction was to exclude false positive fraction (P3, bottom right), and was sorted into 96 well plates for single-nucleus multi-omics experiment.

We collected 36 frozen tumor samples with detailed clinical metadata, resected in three hospitals (Fig. 1a,b); 32 matched samples were collected from 15 patients (11 IDH-mutant astrocytoma (IDH-A) and four IDH-mutant oligodendroglioma (IDH-O), while the remaining four tumors were non-matched primary or recurrent specimens (Fig. 1b and Supplementary Table 1). Most grade 3 or 4 tumors (high grade) received radiation and alkylating agent therapy after surgical resection (Fig. 1b and Supplementary Table 1). Each sample was profiled by bulk whole-exome sequencing (WES), 3′-end snRNA-seq (10x Genomics Chromium) for high-throughput expression data and joint multimodal single-nucleus profiling by SS2 and XRBS (Extended Data Fig. 1a,b).

**Extended Data Fig. 2:**
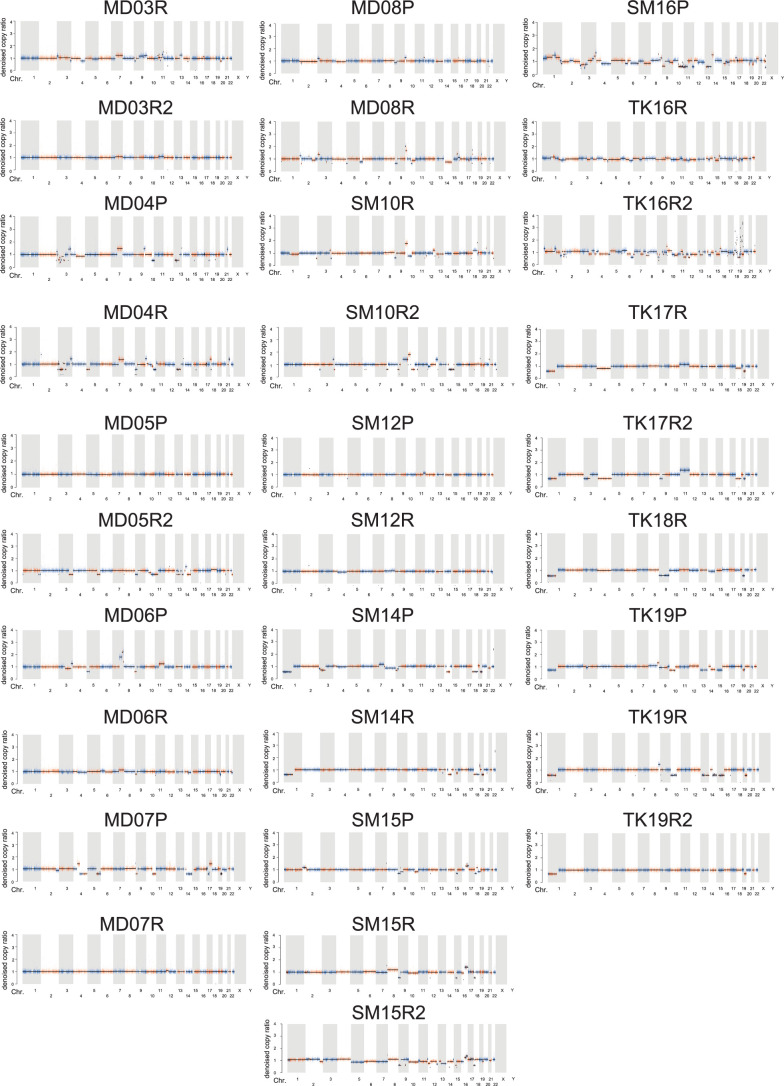
CNAs inferred by WES data. CNAs were inferred from WES data using GATK-CNV pipeline (GATK v4.1.2.0).

**Extended Data Fig. 3:**
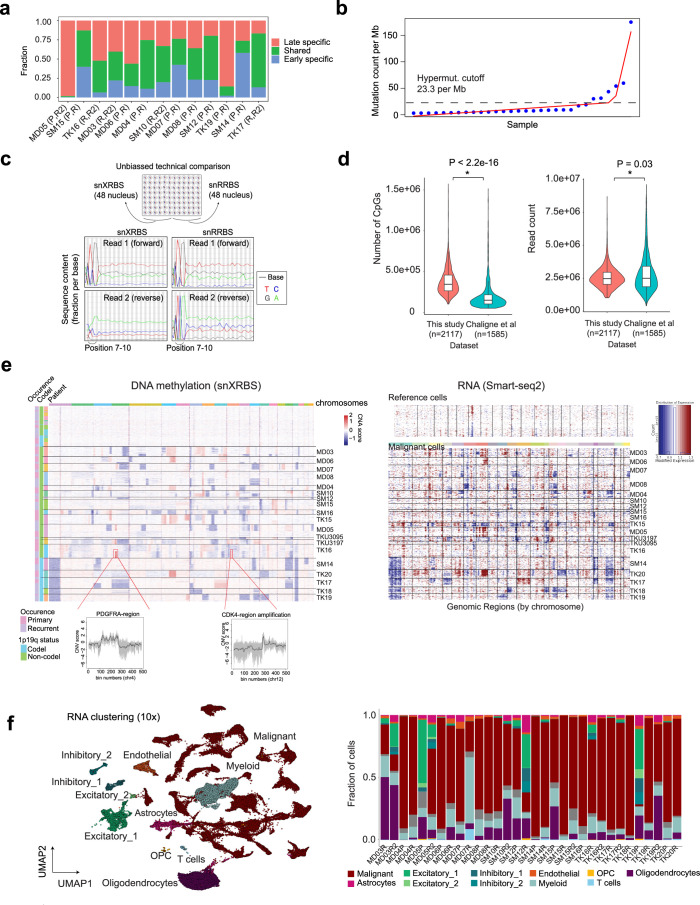
Genetic analysis and technical comparisons within the dataset. **a**, Total fraction of mutations that are either detected only in early timepoint, only in late timepoint or across both early and late timepoints in WES data. **b**, Segmented linear regression analysis was used to determine the cutoff for hypermutators (Hypermut.) as a previous report^51^. This method revealed the presence of a cutoff of a breakpoint at 23.3 mutations per Mb in our dataset**. c**, Top: Schematic for unbiased technical comparison between snXRBS and scRRBS. To remove the sample quality bias and experimental batch effect, we compared two groups within a plate. All processes before the random hexamer steps were done together for both groups. Bottom: Per base sequence content plot for snXRBS (left) and snRRBS (right) data. While the barcode sequences are confirmed on both ends in snRRBS data (Read 1 position 7-10; T-T(C if methylated)-G-G, in Read 2 position 7-10; T-C-A-A), which reflect MspI digestion site, snXRBS lacked it on Read 2 due to the random hexamer priming. **d**, Left: Comparing number of unique CpGs in this snXRBS study and in our previous scRRBS study. Right: Number of aligned reads in this snXRBS study and in our previous snRRBS study. Statistical significance was estimated using a two-sided Mann-Whitney *U* test. Violin plots show the distribution of G-CIMP scores per sample, while box plots indicate the median, interquartile range (IQR), and whiskers extending to 1.5 × IQR. **e**, Heatmap of CNA profiles from DNA methylation data and CNA profiles from RNA-seq data for all samples. CNA score for DNA methylation based on log2 fold-change of read counts in genomic bins compared to mean of non-malignant cells. RNA-seq CNA profiles based on inferCNV with window length of 200. CNA profiles for the genomic region around *PDGFRA* and *CDK4* focal amplifications in TK16R malignant cells (100 kb bins). **f**, UMAP visualization of clustering of snRNA-seq 10X data based on transcriptional profiles (left panel). Cell types were identified based on correlation with normal brain gene expression profiles. Bar plot of proportion of cell types in each tumor (right panel). Excitatory, excitatory neuron; Inhibitory, inhibitory neuron; OPC, oligo-progenitor cell.

Mutation and CNA analysis from bulk WES data showed that most pairs acquired additional genomic alterations upon recurrence (Fig. 1c bottom). Although most cases display shared CNA patterns, in some instances, CNA patterns may diverge, especially in some IDH-A cases. We attribute this pattern to sampling and the lack of canonical CNA events in IDH-A. However, even in those cases, many single nucleotide variant mutations were shared, supporting a common ancestor and a phylogenetic process (Extended Data Figs. 2 and 3a). Four out of 12 pairs acquired *CDKN2A* homozygous deletion, but other acquired events were variable between patients, highlighting the diverse genomic evolutionary trajectories of IDH-G progression (Fig. 1c bottom). Mutational burden analysis showed a drastic increase in the number of C to T alterations with acquired mismatch-repair gene alterations following temozolomide treatment in two cases (MD05R and TK19R) (Fig. 1c top and Extended Data Fig. 3b). In addition to these temozolomide-induced hypermutators, we identified four de novo hypermutated tumors (two pairs) unrelated to treatment (Fig. 1c top and Extended Data Fig. 3b)^27–29^.

Direct comparison of snXRBS with single-nucleus reduced-representation bisulfite sequencing (snRRBS) demonstrated that snXRBS achieved higher coverage in non-promoter regions (*P* = 2.2 × 10^−16^, Mann–Whitney *U*-test; Fig. 1d right and Extended Data Fig. 3c) as well as in promoter regions (*P* = 3.4 ×10^−12^, Mann–Whitney *U-*test; Fig. 1d left). This superior coverage across different genomic regions is critical, as DNA methylation changes in IDH-G progression occur preferentially in non-promoter regions^23–25^. We profiled one plate (96 cells) per sample for dual sequencing (*n* = 36 samples), then included cells that passed our quality control metrics (Methods), both for DNA methylation and transcriptome, retaining a mean of 58.8 (range, 24–85) cells per sample. We also retained cells profiled by snXRBS from one sample (TKU3095) that was used for snXRBS and snRRBS comparisons (Fig. 1d). snXRBS captured a mean (±s.e.m.) of 378,888 ± 3,587 unique CpGs per nucleus (Extended Data Fig. 3d and Supplementary Table 2), higher than the number of unique CpGs profiled by scRRBS in a prior study by our group (mean ± s.e.m., 198,345.1 ± 4,307, *P* = 2.2 × 10^−16^, Mann–Whitney *U*-test; Extended Data Fig. 3d)^26^. The number of detected genes from the matched transcriptomic data (mean ± s.e.m., 2,987 ± 27 genes per nucleus, 889,254 ± 6,624 reads per nucleus, *n* = 2,117) was comparable to that of a stand-alone snRNA-seq SS2 dataset we recently published (mean ± s.e.m., 3,124 ± 13 genes per nucleus, 966,210 ± 7,170 reads per nucleus, *n* = 7264)^31,32^.

We leveraged this dataset to first classify cells as malignant or non-malignant based on clustering of gene expression profiles and DNA methylation (100 kb bins), and validated our classification based on CNA events (Fig. 1e, Extended Data Fig. 3e and Methods). snXRBS-based CNA inference provided greater resolution than snRNA-seq and enabled detection of focal amplification of oncogenes (for example, *PDGFRA* and *CDK4* amplification in TK16R2) (Extended Data Fig. 3e), orthogonally validated by WES (Extended Data Fig. 2). For larger-scale profiling of cellular diversity, we complemented our dataset with 10x 3′-end snRNA-seq in *n* = 32 samples, retaining a total of 158,143 nuclei that passed quality control (Methods). Integrated clustering was performed using Seurat across all samples^33^. We identified non-malignant cell types based on their signatures and correlations with snRNA-seq brain datasets (Extended Data Fig. 3f). Malignant cells were identified based on the presence of CNAs or co-clustering with other malignant cells (Methods).

### Interrogating DNA methylation loss in single cells

**Fig. 2:**
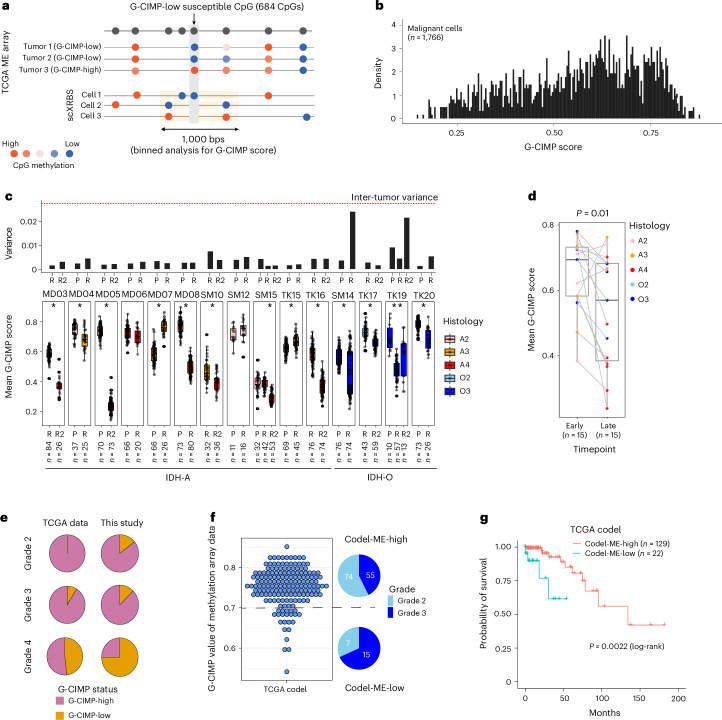
G-CIMP score measured at the single-cell level in IDH-mutant glioma progression. **a**, Schematic of the approach to define the G-CIMP score with snXRBS data. ME, methylation. Red and blue dots represent methylated and unmethylated CpGs, respectively; 1,000 bp windows were created around 684 CpGs that showed lower methylation values in G-CIMP-low progressive samples in a previous study^23^. The average methylation value of all CpGs in all 684 windows was defined as the G-CIMP score in this study. **b**, Histogram of G-CIMP scores across malignant cells from all tumors. **c**, G-CIMP scores for each single nucleus annotated to malignant cells across samples. Each dot reflects a nucleus. The number of dots per sample is indicated in the figure. Boxplots show the median and IQR, and whiskers extend to 1.5× the IQR for G-CIMP scores per sample. The color of the box reflects the pathological subgroup and grade. G-CIMP scores were compared between timepoints (P, primary; R, recurrence; R2, 2^nd^ recurrence) within each patient, using a two-sided Mann–Whitney *U-*test; *P < 0.05. Top panel, barplot of intra-tumor variation of G-CIMP score across malignant cells (red line indicates variance across all tumors). A2, IDH-A grade 2; A3, IDH-A grade 3; A4, IDH-A grade 4; O2, IDH-O grade 2; O3, IDH-O grade 3. **d**, Sample level average G-CIMP scores of malignant cells comparing between early timepoint and late timepoint. Each dot reflects a sample. The color of the dot reflects the pathological subgroup and grade. Boxplots show the median and IQR, and whiskers extend to 1.5× the IQR for G-CIMP scores per timepoint. *P* values represent two-sided paired Wilcoxon rank-sum test. **e**, Pie charts show the proportion of G-CIMP-high samples and G-CIMP-low samples per grade for IDH-A. Left panels show the proportion in a TCGA study^24^, defined based on the bulk DNA methylation array analysis. The right panels show the fraction in our cohort, defined based on the average G-CIMP score per patient inferred by snXRBS analysis. **f**, Dot and boxplots show the G-CIMP score for IDH-O (‘codel’) samples in a TCGA study. The IDH-O samples were divided into two groups (‘codel-ME (methylation)-high’ and ‘codel-ME-low’) based on the score. **g**, Kaplan–Meier curve depicting the overall survival time according to G-CIMP score for the TCGA IDH-O samples. Statistical significance of the survival difference between the groups in each panel was computed using the log-rank test.

**Extended Data Fig. 4:**
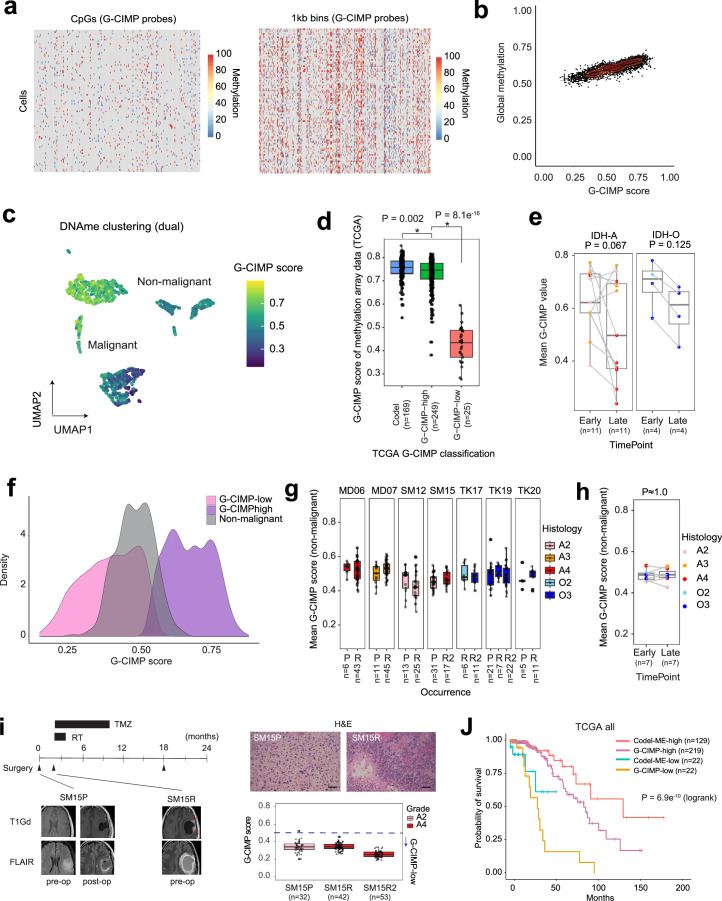
G-CIMP score measured in single cell during IDH-G progression and implications for clinical outcome. **a**, Heatmap of DNA methylation in MD01P for 684 CpGs that are G-CIMP-low susceptible loci. Rows represent individual cells, columns indicate individual CpGs (left) or 1 kb bins surrounding CpGs (right). Color reflects methylation level, gray denotes missing values. **b**, Correlation between G-CIMP score and global methylation based on mean methylation across all CpG sites captured. **c**, UMAP visualization of DNA methylation clustering based on mean methylation in 100 kb bins. Cells colored based on G-CIMP score. **d**, G-CIMP scores inferred for IDH-A (“G-CIMP-high” and “G-CIMP-low”) and IDH-O (“codel”) samples in a TCGA study by Ceccarelli et al. The G-CIMP score was inferred from bulk DNA methylation array data. **e**, Sample level G-CIMP scores comparing between time points, separated by IDH-G subgroup. The color of the dot reflects the pathological subgroup and grade as in g and h. **f**, Density plot of G-CIMP scores in G-CIMP-low and -high cells and non-malignant cells. **g**, G-CIMP scores for each single nucleus annotated to non-malignant cells across samples. **h**, Sample level average G-CIMP scores of non-malignant cells comparing between time points. **i**, A representative case (SM15) showing the discrepancy between pathological diagnosis and G-CIMP score. Left panel shows the clinical course and MRI images. Right top panel shows the hematoxylin and eosin (H&E) images (scale bar, 50μm). Right bottom panel shows the G-CIMP score inferred with the snXRBS data. A dashed line indicates G-CIMP-low cutoff. The primary tumor was not enhanced by gadolinium. Pathologically diagnosed as grade 2, but the G-CIMP score on the snXRBS data classified it as G-CIMP-low aggressive tumor. The tumor recurred shorty with gadolinium enhancement on MRI image and was diagnosed as grade 4. **J**, Kaplan-Meier curve depicting the overall survival time for TCGA IDH-A (“G-CIMP-low” and “G-CIMP-high”) and IDH-O samples (“Codel”). “Codel” group was further divided into “Codel-ME-high” and “Codel-ME-low” based on the G-CIMP score. Box plots show the median, interquartile range (IQR), and whiskers extending to 1.5 × IQR (**d, e, g, h, i**). P-values were determined by a two-sided Mann-Whitney *U* test (**d, g**), a two-sided paired Wilcoxon rank sum test (**e, h**) and a log-rank test (**J**).

We focused on a set of CpG sites (*n* = 684) that were previously identified in bulk studies as differentially methylated during IDH-G progression^23^ and that defined G-CIMP-low IDH-G tumors. To address coverage sparsity, we used genomic bins to compare methylation levels across cells, as previously reported^26^. As a quantitative measure of progression from G-CIMP-high to G-CIMP-low, we assigned a ‘G-CIMP score’ per cell based on the mean DNA methylation across CpGs captured within 1,000 bp bins around each of these 684 CpG sites susceptible to methylation loss. (Fig. 2a, Extended Data Fig. 4a, Supplementary Table 2 and Methods). We calculated the G-CIMP score for malignant cells and classified individual cells as G-CIMP-high or G-CIMP-low using a two-state mixture model (Fig. 2b). The G-CIMP score correlated with global DNA methylation level (*R* = 0.81) but had higher variability, consistent with preferentially capturing loci that are differentially methylated during IDH-G progression (Extended Data Fig. 4b). Additionally, clustering based on mean DNA methylation of 100 kb bins also separated malignant cells primarily based on the G-CIMP score (Extended Data Fig. 4c). When computing this score in bulk DNA methylation arrays from The Cancer Genome Atlas (TCGA), G-CIMP-low samples showed a significantly lower score than G-CIMP-high samples (*P* = 8.1 × 10^−16^, Mann–Whitney *U-*test), confirming that we recapitulated TCGA G-CIMP tumor classification (Extended Data Fig. 4d).

We leveraged our G-CIMP score to assess inter-tumor and intra-tumor heterogeneity of G-CIMP status. We observed that the mean G-CIMP score across malignant cells per tumor is significantly decreased at recurrence (*P* = 0.01, Mann–Whitney *U*-test; Fig. 2c,d and Extended Data Fig. 4e), consistent with expectations from prior bulk studies^21,23,25^. Additionally, the fraction of G-CIMP-high and G-CIMP-low tumors as a function of tumor grade is similar in our cohort and in the TCGA cohort (Fig. 2e)^24^. Importantly, the G-CIMP score in non-malignant cells was stable across timepoints, supporting that the variability observed in malignant cells is not driven by technical factors (Extended Data Fig. 4f–h). We wondered whether the decrease in DNA methylation in malignant cells across timepoints is uniform across cells in a given sample or is driven by a subset of cells. We observed that in most tumors, the G-CIMP score has low variance across malignant cells, suggesting that G-CIMP status is similar between malignant cells within a tumor sample (Fig. 2c).

Although our observations were concordant with prior studies, we detected a few outliers in our cohort: a G-CIMP-low grade 2 IDH-A (Fig. 2e) that notably displayed a very aggressive clinical phenotype (Extended Data Fig. 4i), and two recurring IDH-O tumors as G-CIMP-low (Fig. 2c,d), an unexpected observation given that prior studies had limited the G-CIMP-low phenotype to IDH-A^24^. In a reanalysis of the TCGA cohort by G-CIMP score, we detected subsets of IDH-O with relatively lower G-CIMP scores (<0.7) (Fig. 2f). These tumors were enriched in grade 3 tumors (Fig. 2f) and showed significantly worse overall survival (*P* = 0.0022, log-rank; Fig. 2g). Moreover, these IDH-O with low methylation showed worse prognosis than IDH-A with high methylation (G-CIMP-high vs codel-ME-low, *P* = 0.0143; Extended Data Fig. 4j). These results suggest that hypomethylation is associated in both IDH-A and IDH-O with recurrence and a more aggressive clinical trajectory and may serve as an additional prognostic marker across all IDH-G subsets^34^.

### Hypomethylation, increase in stem-like states in progressed IDH-G

**Fig. 3:**
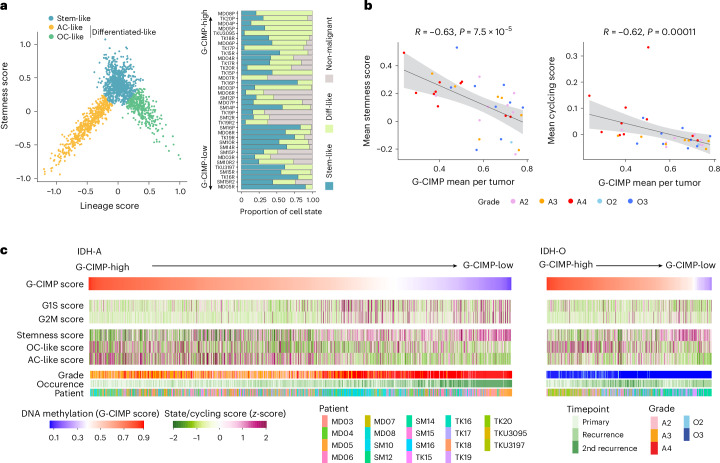
G-CIMP loss reshapes cell states hierarchies. **a**, Left, lineage plot based on transcriptome profiles of dual protocol data. Cells were classified as stem-like, AC-like or OC-like based on a previously described method^17^. Right, bar plot of proportion of stem-like, differentiated-like (AC-like and OC-like) and non-malignant cells per tumor. **b**, Scatter plots showing the proportions of stem-like cells (left) and cycling cells (right) versus the mean G-CIMP score. Each dot represents a sample. Color reflects pathological grade and subgroup. Shown is the Pearson’s correlation coefficient and associated *P* value; shaded area, confidence intervals. **c**, IDH-A (top) and IDH-O (bottom) cells (columns) were ordered by the G-CIMP score calculated by XRBS data. Cycling scores (G1S and G2M) and cell state scores (stemness, AC-like and OC-like) inferred by SS2 were shown. Additional rows were for the clinical information (patient, occurrence and pathological grade) of the samples from which the cells were derived.

**Extended Data Fig. 5:**
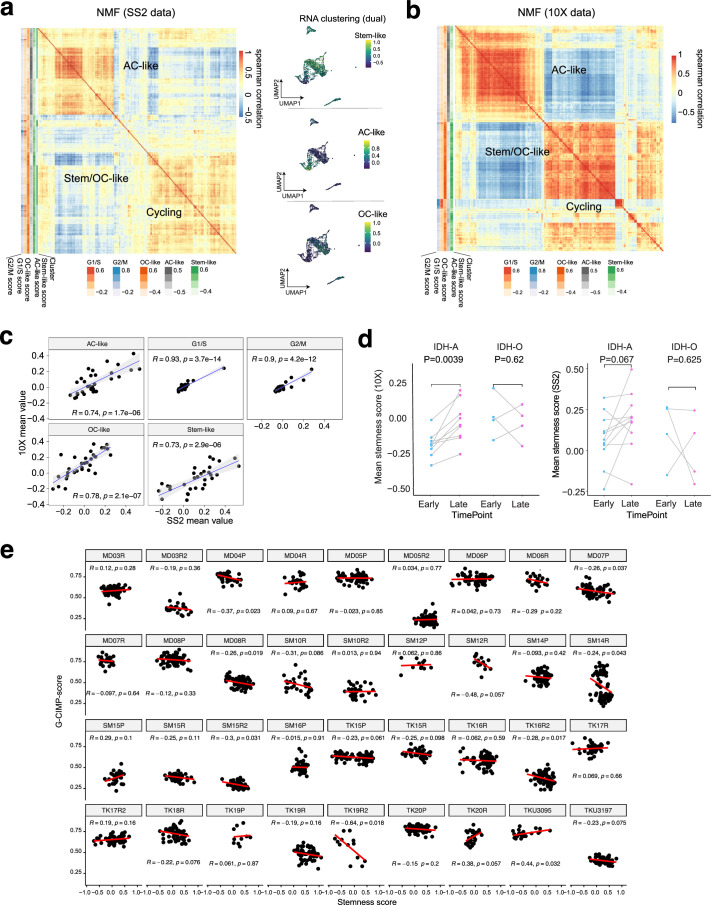
*De novo* NMF of IDH-G states and changes associated with decreased G-CIMP status. **a**, Correlation matrix of module score across all cells based on non-negative matrix factorization (NMF) programs defined in each sample (left panel). Row annotations based on correlation of each program with scores from previously defined cell state gene signatures. Three major programs were detected that correlated highly with AC-like, Stem/OC-like and cycling. Clustering of cells based on transcriptome profiles and cells colored based on module score for the three malignant cells states (right panel). **b**, The same NMF analysis as in Extended Data Fig. 3a was also performed on the SS2 dataset, identifying similar programs. **c**. Scatter plot of mean module score expression of malignant cells between SS2 and 10X based on IDH-G cellular state defined in our previous paper^17^. Each dot reflects a sample and the shaded area is based on confidence interval and spearman correlation and associated two-sided p-value shown. The samples having both SS2 data and 10X data were included in this analysis. **d**, Paired comparison of proportion of stem-like cells in primary versus recurrent tumors based on 10X data (left, n = 9 for IDH-A and n = 4 for IDH-O) and SS2 (right, n = 11 for IDH-A and n = 4 for IDH-O). P-values represent two-sided paired Wilcoxon rank sum test. **e**, Scatter plots show the stem-like program (x axis) versus G-CIMP score (y axis). Each dot represents a cell. Panels are separated by sample. Pearson’s r and associated two-sided p-value are shown.

We and others have previously leveraged scRNA-seq to characterize the cellular composition and putative differentiation trajectories of IDH-G^15,19^. Our studies identified three major compartments consisting of stem-like states enriched in cycling cells and more differentiated cells along either the AC-like or OC-like lineages^15,17,19^. Given technical differences in tissue processing and profiling (snRNA-seq vs scRNA-seq), we first tested whether an unbiased analysis of our dataset would recapitulate the previously described signatures. We applied non-negative matrix factorization to expression data from 10x and SS2 datasets (Extended Data Fig. 5a,b) and derived robust expression programs that were consistently detected across multiple parameters, as previously described^35,36^. Consistent with our prior studies, we identified major clusters of programs that correlated with the AC-like state, stem-like or OC-like state and a cycling state (Extended Data Fig. 5a and Methods) and classified malignant cell states accordingly in both our 10x and SS2 datasets (Fig. 3a, Extended Data Fig. 5c and Supplementary Table 2)^17,19^. We then assessed the longitudinal changes in state proportion and observed an increase in stem-like cells and a decrease in differentiated-like cells in recurrent tumors (*P* = 0.039) (Extended Data Fig. 5d), consistent with prior scRNA-seq studies performed in non-matched samples^15,19^.

Our results so far indicate that IDH-G progression and transition to G-CIMP-low is associated transcriptionally with an increase in stem-like cells (*P* = 3.4 × 10^−4^, *R* = −0.56; Fig. 3b left) and cycling cells (*P* = 0.0001, *R* = −0.62; Fig. 3b right), and epigenetically with a decrease in G-CIMP score (Fig. 3c). We therefore generated two hypotheses: glioma stem-like cells display a lower G-CIMP level and their expansion leads to tumors with overall lower G-CIMP; or tumors that transition to a G-CIMP-low skew cell state composition by hypomethylating regions that are important in cell state determination. In line with our observation of low intra-tumor variance in G-CIMP scores, we observe no correlation between G-CIMP score and stemness score (Extended Data Fig. 5e). This suggests that G-CIMP status is similar in different IDH-G cell states and would not support our first hypothesis. To test our second hypothesis, we performed three independent analyses, described below.

### Putative mechanistic links between methylation loss and increase in stem-like states

**Fig. 4:**
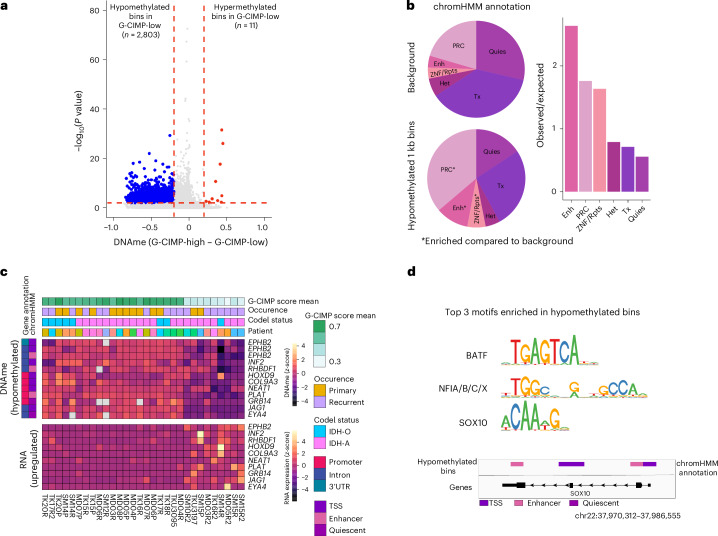
Hypomethylated regions in G-CIMP-low tumors are enriched for stem-like features. **a**, Volcano plot of differentially methylated 1 kb bins between G-CIMP-high and G-CIMP-low cells across all tumors (false discovery rate-adjusted *P* value values based on two-sided Student’s *t*-test). **b**, Annotation of differentially methylated bins based on overlap with chromHMM labels from IDH-G. The *P* value was calculated using a permutation test by comparing the observed overlap between the region of interest and matched random regions (*n* = 1,000) to the null distribution generated from those permutations. Enh, enhancer; PRC, polycomb repressed complex; ZNF/Rpts, zinc finger genes/repeats; Het, heterochromatin; Tx, transcription. **c**, Hypomethylated bins that overlap with upregulated genes in G-CIMP-low tumors compared to G-CIMP-high tumors. Gene features of the bins are shown on the left. UTR, untranslated region. **d**, Top three enriched transcription factor motifs in hypomethylated bins (top panel). Location of 1 kb bins that are hypomethylated across the *SOX10* gene (bottom panel). TSS, transcription start site; chr, chromosome.

**Extended Data Fig. 6:**
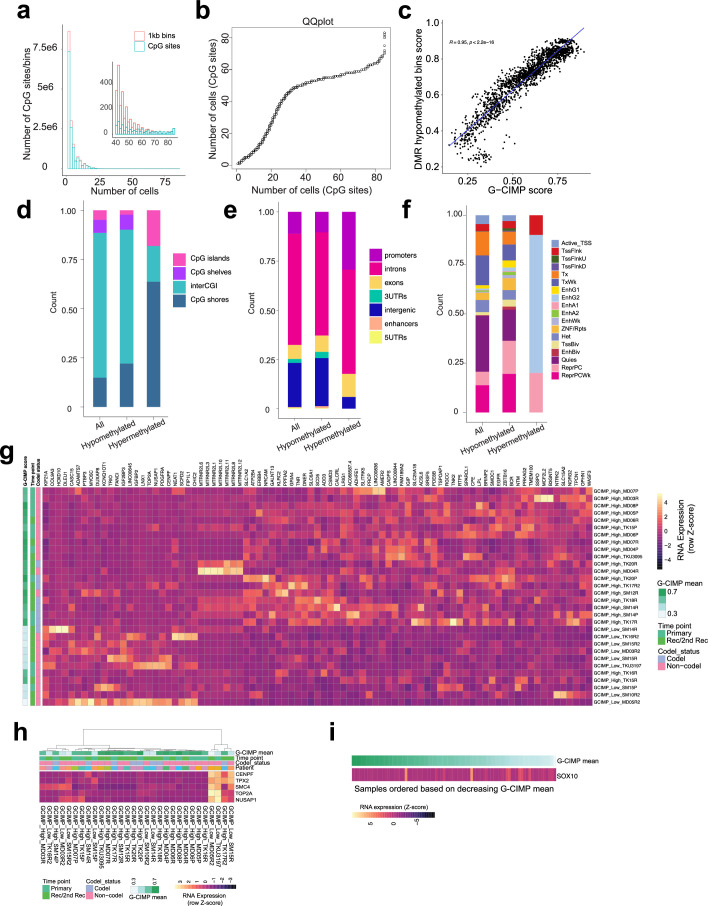
Annotation of differentially methylated bins and differentially expressed genes in G-CIMP high *vs* low cells. **a**, Histogram of the number of cells in sample MD03R where methylation at a CpG site or 1 kb bin is captured. With 1 kb bins, we observe a higher frequency of capture. **b**, QQ-plot to compare the distribution of capture per cell between CpG sites and 1 kb bins. Each point represents the quantiles across the two distributions. **c**, Scatter plot of all malignant cells comparing the G-CIMP score to the mean methylation across 1 kb bins that are hypomethylated in G-CIMP low tumors and spearman correlation and associated two-sided p-value shown. **d–f**, Overlap of differentially methylated 1 kb bins with CpG island annotation from annotatr R package, gene annotation and chromHMM labels from IDH-mutant gliomas. **g**, Differentially expressed genes between G-CIMP high and low cells across all tumors. **h**, Cell cycle related genes that are differentially expressed between G-CIMP high and low tumors. **i**, Mean expression of *SOX10* gene across malignant cells per tumor.

We first focused on DNA methylation and identified genomic regions that are consistently hypomethylated in G-CIMP-low cells across tumors. We generated 1 kb bins to increase the number of regions that could be evaluated across cells (Extended Data Fig. 6a,b) and then compared the mean DNA methylation levels of these regions between G-CIMP-high and G-CIMP-low cells (cells with intermediate G-CIMP scores were excluded; Methods). This approach detected 2,803 hypomethylated bins and 11 hypermethylated bins in G-CIMP-low tumors (Fig. 4a). Scoring cells using this broader set of differentially methylated regions showed a strong correlation with the G-CIMP scores calculated from the previously reported 684 regions (Extended Data Fig. 6c). Consistent with previous studies, we observed that hypomethylated bins are enriched in regions outside of CpG islands and shores (<2 kb from CpG islands) (Extended Data Fig. 6d). Additionally, we also detected several hypomethylated bins within introns (Extended Data Fig. 6e). We annotated hypomethylated bins based on chromHMM labels^37^ from IDH-G tumors and observed an enrichment in PRC-associated domains, enhancers and ZNF/repeat regions (Fig. 4b and Extended Data Fig. 6f). Interestingly, our previous single-cell multi-omics analysis of IDH-wildtype glioblastoma demonstrated hypomethylation of PRC-associated genes in stem-like cells, and suggested that PRC2 may serve as a critical switch in glioma cell differentiation^26^. This observation is consistent with studies demonstrating the role of PRC2 in glioma stem-like cells and further supports the parallels between glioma differentiation and neurodevelopment^38,39^. Therefore, in G-CIMP-low tumors, the decrease in methylation may preferentially lead to PRC2 target hypomethylation, which may help preserve IDH-G stemness potential.

As a second analysis, we leveraged the use of the multimodal protocol to associate gene expression changes with DNA methylation. We first performed differential gene expression analysis between G-CIMP-high and G-CIMP-low tumors (Extended Data Fig. 6g and Supplementary Table 3–4). We observed upregulation of known stemness genes, such as *HOXD10*, *HOXD9* and *PROM1*, in G-CIMP-low tumors, which are potential PRC2 targets^40–42^. Additionally, we observed a subset of cycling genes that were upregulated in G-CIMP-low tumors (Extended Data Fig. 6h). Next, we assessed the overlap between hypomethylated promoters and changes in gene expression between G-CIMP-high and G-CIMP-low cells. Importantly, among the differentially expressed genes, only a limited number showed decreased promoter methylation; these comprised 11 genes, including *HOXD9* and *EPHB2*, potential mediators of glioma progression (Fig. 4c)^43–45^. There can be several explanations for this discrepancy. First, enhancers are particularly susceptible to hypermethylation in IDH-G and may contribute to gene expression dysregulation^26^. Second, stochastic epimutations may accumulate in cancer, leading to a decoupling of the anti-correlation between gene expression and promoter methylation^26^. Third, hypermethylation of CTCF binding sites may cause aberrant gene activation through loss of gene insulation^9^. Previous studies based on bulk assays have also demonstrated a weak association between DNA methylation and transcriptional alterations in gliomas and attribute changes primarily to changes in PRC-dependent H3K27me3 (ref. ^43^). Therefore, transcriptional deregulation in G-CIMP-low tumors may result from direct hypomethylation of cognate gene promoters or from indirect expression dysregulation.

Finally, as a third analysis, we interrogated hypomethylated regions for enrichment in transcription factor binding motifs, as methylation loss may affect transcription factor binding^46,47^. Motif analysis of hypomethylated bins showed enrichment for binding motifs of BATF, the NFI family and SOX10, a master regulator of glioma lineages (Fig. 4d)^37^. Of note, *SOX10* itself is hypomethylated and moderately increased in expression in a subset of G-CIMP-low tumors (Extended Data Fig. 6i). Collectively, our multimodal single-cell analysis does not provide support for the hypothesis that intercellular variability in G-CIMP status underlies DNA methylation changes of IDH-G progression; rather, the data show that G-CIMP status is stable between different cell states of the same tumor. However, we identify three putative mechanistic links between DNA methylation loss and an increase in stem-like cell state: hypomethylation of PRC2 targets; the increased expression of stem-cell genes; and the enrichment in binding motifs of key stem-cell transcription factors. These observations lend support to the hypothesis that G-CIMP-low tumors may skew the cellular state composition by hypomethylating regions that are important in cell state determination.

### Increased heritability of stem-like state in IDH-G progression

**Fig. 5:**
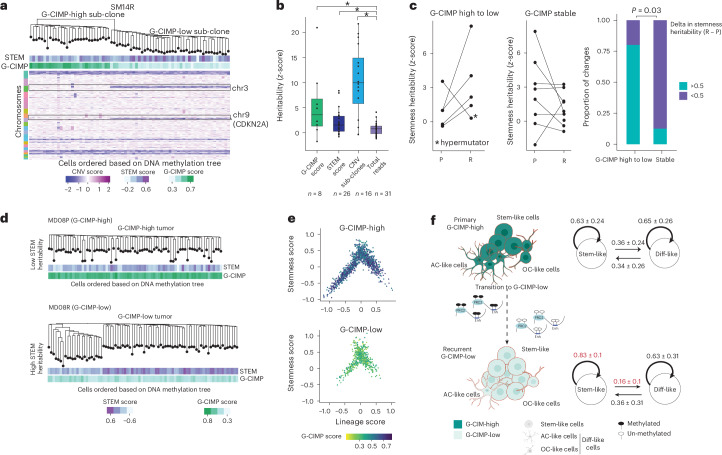
High-resolution DNA methylation lineage trees enable the assessment of cell state dynamics in G-CIMP-high versus G-CIMP-low tumors. **a**, Lineage tree based on DNA methylation sites across the genome in the SM14R tumor. Annotation of cells includes stemness score, G-CIMP score and CNA profiles based on DNA methylation data. **b**, Heritability score based on DNA methylation lineage trees per tumor (each point in boxplot). For each feature, tumors with sufficient heterogeneity were used to ensure that heritability could be measured (Methods). Significance between G-CIMP score, stemness score and CNA compared to total reads based on a Fisher’s exact test for the number of samples with a *z*-score of >2. Boxplot spans the IQR, with the median line displayed and the whiskers extending 1.5× the IQR. STEM, stemness. **c**, Paired analysis comparing the heritability of the stemness score between primary and recurrent tumors from the same patient. Patients with a difference of less than 0.3 in the G-CIMP score between primary and recurrent were classified as G-CIMP stable. Fisher’s test was performed based on increase or a decrease/no change in heritability (no change defined as delta heritability less than 0.5). **d**, Example of a DNA methylation lineage tree in which the stemness score is more heritable in the recurrent G-CIMP-low tumor compared to the primary G-CIMP-high tumor. **e**, Lineage plots of all malignant cells separated based on G-CIMP categories. Cells are colored based on the G-CIMP score. **f**, Overall model of G-CIMP-high to G-CIMP-low transition that is accompanied by changes in cell state proportions (mean values of cell state transition probabilities displayed in right panels; error bars, s.d.). Diff-like, differentiated-like.

**Extended Data Fig. 7:**
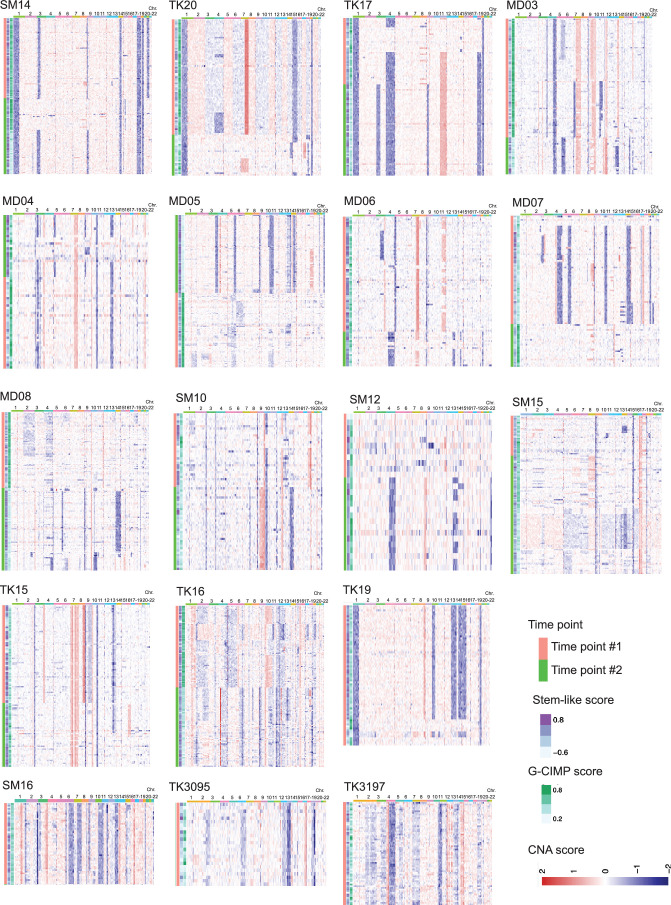
Copy number profiles based on DNA methylation across all tumors. Heatmap of copy number profiles of malignant cells only for each patient (primary and recurrent) based on the ratio of mean DNA methylation in 2MB bins per cell to the mean of all non-malignant cells. Cells are also annotated based on G-CIMP score and stemness score from corresponding RNA data. TK19, SM16, TK3095, TK3197 include single timepoint. TK18 is not shown, as no CNA was detected.

Malignant cells in gliomas display a spectrum of hierarchical versus plastic organization^26^, but elucidating their precise organization in clinical samples remains challenging. Given the increase in stem-like cells in high-grade IDH-G, we reasoned that tumor progression may lead to differences in cell state transition dynamics. To address this possibility, we leveraged phylodynamics and characterized the lineage organization of malignant cells within each tumor, comparing G-CIMP-low versus G-CIMP-high samples. First, we constructed phylogenetic trees for each tumor based on DNA methylation epimutations as previously described^26,30^. We validated the trees using CNAs and observed that sub-clonal populations defined solely based on CNAs aligned to different clades on the trees constructed from epimutations (Fig. 5a and Extended Data Fig. 7).

We then used the Phylogenetic Analysis of Trait Heritability (PATH) framework to calculate the heritability versus plasticity of malignant cell states^48^. PATH quantifies the extent to which cells cluster by phenotype or state on a phylogeny with phylogenetic correlations and transforms these measurements into inferences of cell state transition dynamics. We applied PATH to our cohort and first assessed the heritability of the G-CIMP score and stemness score in tumors with sufficient heterogeneity of these features. As expected, we observed that the G-CIMP score and stemness score are each heritable within a tumor, with significant clustering on the phylogenetic tree, as compared to non-heritable programs such as total reads (Fig. 5a,b). Next, we performed a paired analysis of matched primary and recurrent tumors that differ in G-CIMP status and observed an increase in heritability of stem-like state in four out of five patients that transitioned from G-CIMP-high to G-CIMP-low (Fig. 5c). The only outlier (MD05) was a hypermutated case with 95% of the cells being assigned as stem-like, reducing our ability to accurately quantify heritability based on tree distribution of cell states.

**Extended Data Fig. 8:**
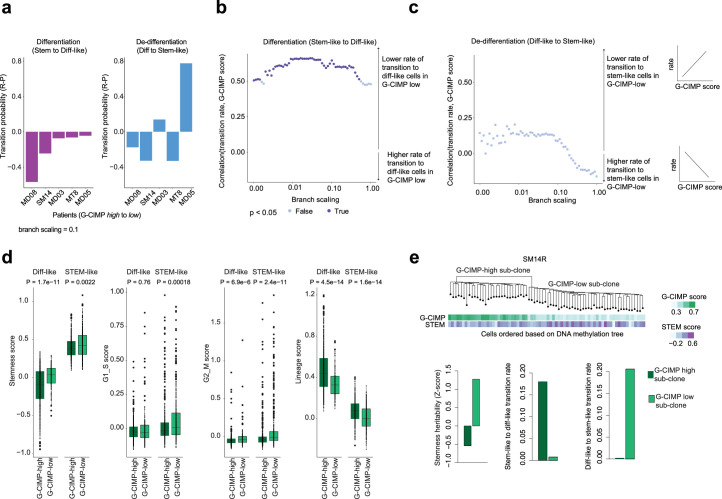
Changes in cell state transition dynamics in G-CIMP high *vs* low tumors. **a**, Bar plot of difference in transition probability between primary and recurrent tumors. **b–c**, Correlation between cell state transition rate and G-CIMP score per tumor across different branch scaling values. Higher correlation represents lower rate in G-CIMP low compared to G-CIMP high. Spearman’s rank correlation between cell state transition rate and G-CIMP score per tumor across different branch scaling values; statistical significance was measured with a two-sided test. **d**, Boxplot of cell state scores between G-CIMP high (n = 506 for Diff-like and n = 305 for Stem-like) and low cells (n = 108 for Diff-like and n = 238 for Stem-like). Box plot spans the interquartile range (IQR) with the median line displayed and the whiskers extend by 1.5 x IQR.P-value calculated based on two-sided Student’s t-test. **e**, Lineage tree for SM14R sample based on DNA methylation reveals two major sub-clones based on G-CIMP score (top panel). Heritability score and cell state transition rates for each G-CIMP high and low sub-clone shows values similar to the trend observed across tumors (bottom panels).

Importantly, PATH quantifies cell state transition dynamics by measuring how frequently different transcriptional states occur phylogenetically adjacent to one another. We first applied PATH to five patients with matched primary and recurrent tumors that became G-CIMP-low, allowing us to assess how cellular transitions change with tumor progression while controlling for inter-patient variability. In all five cases, recurrent tumors showed a lower probability of transitioning from stem-like to differentiated-like states than the matched primary tumors, indicating reduced differentiation in G-CIMP-low recurrences (Extended Data Fig. 8a). To evaluate this relationship more broadly, we next assessed the association between PATH-inferred differentiation and de-differentiation rates and tumor G-CIMP scores across a larger cohort of unmatched tumors, using correlation analysis. Differentiation rates showed a significant correlation with G-CIMP score, with G-CIMP-low tumors consistently exhibiting diminished stem-cell differentiation, whereas de-differentiation rates showed weaker and less consistent associations (Extended Data Fig. 8b,c and Methods). This can also be observed based on the organization of cells along the phylogenetic tree for patient MD08 that transitions from G-CIMP-high to G-CIMP-low, in which we observed a more structured organization of stem-like cells in the recurrent G-CIMP-low tumor (Fig. 5d). Interestingly, we also observed that differentiated-like cells in G-CIMP-low tumors express higher stemness scores, are cycling at higher rate and express lower levels of lineage differentiation scores (Fig. 5e and Extended Data Fig. 8d). This suggests that both the frequency of differentiation and the extent to which differentiation programs are fully deployed in IDH-G cells is impacted by G-CIMP status. We finally leveraged SM14R, a tumor in which we observe distinct G-CIMP sub-clones, to investigate the impact of G-CIMP status on cell state dynamics within a single tumor (Fig. 5a). When calculating heritability for the G-CIMP sub-clones in the SM14R tumor, we confirmed an increase in heritability of stemness in the G-CIMP-low sub-clone compared to G-CIMP-high (Extended Data Fig. 7e). Collectively, our findings support a model in which cell state dynamics are disrupted in G-CIMP-low tumors, with fewer transitions towards differentiated-like cells, higher cell cycle rate and retention of malignant cells in a stem-like state, leading to an increase in proportion of stem-like cells (Fig. 5f).

## Discussion

IDH-G progression is associated with genetic, transcriptional and epigenetic alterations that remain poorly understood^13,21–23,25^. We posited that multimodal profiling in single cells in a progressive IDH-G cohort would enable us to integrate across these perspectives and capture the multi-dimensional evolution of the disease. Transcriptionally, we observe a consistent shift in glioma cell states toward increased proliferation, increased pool of stem-like cells and reduced glioma differentiation as the disease advances, consistent with recent reports in unmatched cohorts^15,19^. This consistent transcriptional trajectory in IDH-G differs from the highly diverse evolutionary trajectories we recently demonstrated in IDH-wildtype glioblastoma^31,32^. Epigenetically, we provide single-cell evidence of recurrence-associated hypomethylation, previously reported in bulk analyses^21–25^. Our single-cell analysis empowers the assessment of cancer-cell-specific G-CIMP methylation levels with greater sensitivity, without the confounding effects of the tumor micro-environment.

We wondered what drives changes in cell states as IDH-G progresses. Our multimodality sequencing technology allowed the analysis of methylation–transcription relationships, revealing aberrant epigenetic patterns of IDH-G progression. We identified three putative mechanistic links between DNA methylation loss and the increase in stem-like cell observed in IDH-G progression: (1) the hypomethylation of PRC2 targets, which may allow the rapid reactivation of their expression in response to stimuli, providing a key mechanism for stemness maintenance; (2) the increased expression of glioma stem-cell genes such as *SOX10*, a canonical glioma stem-like marker; and (3) the enrichment in binding motifs of stem-cell transcription factors that may enhance transcription factor genome accessibility. These observations lend support to the hypothesis that the cell state composition may be skewed by hypomethylation of regions important in cell state determination.

The observed parallels between IDH-G differentiation and neurodevelopment invoke the question of whether gliomas follow unidirectional differentiation patterns or reversible bidirectional cell state transitions. Prior work from our groups showed that malignant cells in low-grade IDH-G display a hierarchical organization in which differentiation far outpaces de-differentiation, with only limited bidirectional plasticity^26^; however, how this evolves in IDH-G progression is uncharted. In this study, we leveraged phylodynamics and characterized the lineage organization of malignant cells within each tumor. We observed that cell state dynamics are disrupted in G-CIMP-low tumors, with fewer transitions towards differentiation, higher cell cycle rate and retention of malignant cells in a stem-like state, leading to an increase in the proportion of stem-like cells. Differentially demethylated regions in G-CIMP-low cells may also allow them to reactivate their stemness program and may empower positive selection to enhance the evolutionary capacity of IDH-G, potentially explaining the more aggressive clinical behavior of G-CIMP-low tumors. Importantly, our study demonstrates that G-CIMP status may serve as an important prognostic marker across all IDH-G subsets, extending the concept outside of IDH-A^21,23–25^, as also supported by very recent reports^34^. Additionally, these changes in hierarchies and cell state heritability during IDH-G progression may impact response to mutant IDH inhibitors, as these agents act, at least in part, as differentiation therapies^49^. Future studies should address whether the measurement of G-CIMP methylation levels has a role in stratifying patients for response to mutant IDH inhibitors.

Although our study has deepened our understanding of IDH-G evolution at the cellular level, our work has several limitations. First, snXRBS, although offering increased sampling of CpG, still suffers from the sparsity inherent to single-cell data. We therefore have implemented analytical approaches, including averaging DNA methylation levels across defined genomic windows, to overcome this limitation. Second, despite important efforts to acquire matched clinical cohorts of IDH-G, our cohort is still limited in both the number of samples and number of cells profiled. Third, although snXRBS improves the accuracy of detecting arm-level copy number alterations at the single-cell level, our data remain unsuitable for identifying point mutations in single cells. We envision that future experimental and analytical developments applied to larger cohorts will empower higher throughput and more precise measurement of DNA methylation across the genome in single cells, along with a better understanding of the causal relationship between DNA methylation, mutations and cell states. Our analyses revealed insights into how DNA methylation loss may affect cell state composition and dynamics; however, they also highlight considerable methylation loss that cannot be fully explained by cell state transitions alone. DNA methylation loss related to mitotic age may contribute to G-CIMP hypomethylation, but this could not be directly demonstrated leveraging existing methylation clock signatures^50^.

In conclusion, our study applied concurrent snDNA methylation and transcriptome to the context of IDH-G progression. Although cell state diversity and tumor evolution are often studied independently, the data presented herein show that single-cell multi-omic analysis of progressive IDH-G clinical samples can help draw together these diverse frameworks through the lens of high-resolution phylogenetic trees coupled with annotation for concurrent phenotypic and DNA methylation states. By integrating single-cell methylation and transcription within an evolutionary framework, we show how DNA methylation loss drives stem-like transcriptional states and altered cell state dynamics, offering a blueprint for dissecting evolution in human cancers.

## Methods

### Human subjects and ethical approval

Frozen IDH-G samples were collected from three institutions, listed below. All tumors had the IDH1 R132H mutation, which was confirmed by WES or Sanger sequencing. Collection was approved by the Institutional Review Board of each institution, and all patients provided written informed consent accordingly. Cohorts were added to the Institutional Review Board protocol from the Harvard Cancer Consortium (HCC) 10-417. The tumors were collected from the MD Anderson Cancer Center, approved by the Institutional Review Board of MD Anderson Cancer Center under protocol number 2012-0441. Tumors for the Tokyo University cohort were collected from the Department of Neurosurgery, Tokyo University Hospital, Tokyo, and approved by the Institutional Review Board of Tokyo University Hospital under protocol number G10028. Tumors for the St. Michael’s Hospital cohort were collected from the Division of Neurosurgery, St. Michael’s Hospital, Unity Health Toronto. The samples and data used for the research were obtained from the Unity Health Brain Tissue Biobank and approved by the Research Ethics Board of St. Michael’s Hospital, Unity Health Toronto, under protocol number REB 13-141. Clinical information of the cohorts is summarized in Supplementary Table 1.

### Statistics and reproducibility

No statistical method was used to predetermine sample size. All of the available matched pairs of IDH-G samples were provided by the above centers.

### Nuclei isolation from frozen tissue

Nuclei from frozen tumor tissue were isolated as previously reported^51^. In brief, tumor tissue was thawed and mechanically dissociated in ST buffer (10 mM Tris-HCL pH 7.5, 1 mM CaCl_2_, 146 mM NaCl, 21 mM MgCl_2_) with 0.49% CHAPS (Millipore, 28300). Single-nuclei suspensions were filtered using a 40 μm strainer, centrifuged at 500 g for 5 min and resuspended in ST buffer supplemented with 0.01% BSA (NEB, B9000S). Nucleus suspensions were inspected by microscope, counted using a hemocytometer and used for FACS-sorting for the single-nucleus dual sequencing workflow and for droplet-based 10x snRNA-seq.

### 10x Genomics for single-nucleus sequencing

The 10x Chromium Single Cell 3′ Reagent Kit v3.1 (10x Genomics, PN1000128) was used according to the manufacturer’s protocol. In brief, nuclei were loaded on the Chromium Chip (10x Genomics, PN12000120) with a target cell recovery of 6,000–8,000 nuclei and processed in the Chromium Controller. Single nuclei were partitioned into Gel Beads-in-Emulsion (GEMs), followed by reverse transcription with barcoding. Libraries were created by breaking GEMs and pooling barcoded fractions, cDNA amplification, fragmentation and attachment of sample index and adaptor, and then sequenced on NextSeq 2000 or NovaSeq (Illumina).

### Nucleus sorting for dual single-nucleus sequencing

Nuclear suspension was stained by Vybrant DyeCycle Ruby Stain (Thermo Fisher Scientific, V10309) immediately before FACS sorting. Single-nucleus sorting was performed on a FACS Aria Fusion sorter (Becton Dickinson) using a 640 nm laser (Ruby, 670/14 filter). After discriminating doublets, ruby-positive nuclei were sorted into 96-well plates containing TCL buffer (Qiagen, 1031576) with 1% beta-mercaptoethanol (Sigma-Aldrich, M6250). Plates were frozen on dry ice immediately after sorting and stored at −80°C before single-nucleus dual DNA methylation and transcriptome sequencing.

### Separation of genomic DNA and messenger RNA for the dual single-nucleus experiment

Single nuclei sorted into 96-well plates were profiled by the joint SS2 and XRBS protocol. Genomic DNA (gDNA) and mRNA in 96-well plates were separated as previously described^26,29^. In brief, a modified oligo(dT) primer (Supplementary Table 5) was conjugated to streptavidin-coupled magnetic beads (Dynabeads, Life Technologies) according to the manufacturer’s instructions. To capture polyadenylated messenger RNA (mRNA), 10 μl of conjugated beads (10 μM) was added to the nucleus lysate and incubated for 3 min at 70 °C, then for 20 min at room temperature (20 °C–25 ℃) with mixing to prevent the beads from settling. The mRNA was then collected to the side of the well using a magnet, and the supernatant, containing the gDNA, was transferred to a fresh plate.

### snXRBS

Proteinase K (0.4 U; NEB, P8107S) was added to each well of the gDNA plate, then the gDNA was concentrated on 1× AMPure XP beads (Beckman, A63882). The gDNA was incubated with the restriction enzyme Msp1 (10 U, NEB) for 80 min at 37 °C. Heat-inactivation was performed for 10 min at 70 °C. Digested DNA was filled in and A-tailed at the 3′ sticky ends with 2.5 U of Klenow fragment (NEB, M0212L). The reaction was supplemented with 2 mM dATP, 0.2 mM dCTP and 0.2 mM dGTP (Thermo, R0193) and performed as follows in a thermocycler: 30 °C for 25 min, 37 °C for 25 min and heat-inactivation at 70 °C for 10 min. Custom barcoded methylated adaptors (0.1 μM) were then ligated overnight at 16 °C with the dA-tailed DNA fragments in the presence of 800 U of T4 DNA ligase (800U, NEB) and 1 mM ATP (Roche). T4 DNA ligase heat-inactivation was performed at 70 °C for 15 min the next day. Genomic DNA from 24 individual cells was pooled together according to their barcodes, giving, for a 96-well plate, four pools of 24 cells. Pooled genomic DNA was cleaned up and concentrated using 1.8× AMPure XP beads (Beckman, A63882). Each pool was then sodium-bisulfite-converted (Fast Epitect Bisulfite; Qiagen, 59824). To ensure full bisulfite conversion, two cycles of conversion were performed (98 °C for 5 min; 60 °C for 20 min; 98 °C for 5 min; 60 °C for 20 min). After the thermal cycler step, column wash was performed according to the manufacturer’s protocol, except for replacing the column with Zymo-Spin IC fast-spin columns (ZYMO research, C1004-50). Bisulfite-converted DNA was eluted in 30 μl of TE, then Hexamer extension was performed as previously reported^28^ with a slight modification. Bisulfite-converted single-stranded DNA was mixed with 1× NEB Buffer 2.1, 0.4 mM dNTP mix and 2 μM random hexamer primer (Supplementary Table 5), which was designed to fit our methylated barcode adaptors in this study. The solution was heated at 95 °C for 45 s and then transferred immediately to ice. Then, 1.3 μl of Klenow enzyme (NEB, M0212L) was added to each reaction. Hexamer base pairing is mediated through a gradual increase in temperature from a 4 °C incubation and an incremental increase in temperature to 37 °C at a rate of 1 °C s^−1^ followed by additional incubation at 37 °C for 1.5 h. The product was washed by 1× AMPure XP beads (Beckman, A63882) and was eluted in 30 μl of water, then amplified using primers containing Illumina i7 and i5 index. Following Illumina pooling guidelines, a different i7 index was used for every 24-cell pool, allowing multiplexing of 96 cells: 288 cells for sequencing on one Illumina NextSeq lane. Library enrichment was done using KAPA HiFi Uracil+ master mix (Kapa Biosystems, KK2802), and the following PCR condition was used: 98 °C for 1 min; six cycles of 98 °C for 20 s, 58 °C for 30 s, 72 °C for 1 min; followed by 12 cycles of 98 °C for 20 s, 65 °C for 30 s and 72 °C for 1 min. PCR was terminated by an incubation at 72 °C for 3 min. Enriched libraries were cleaned up and concentrated using 1× AMPure XP beads (Beckman, A63882). Every 24-cell pool was mixed with the other pools in an equimolar ratio. All cells from three 96-well plates were sequenced by a NextSeq 2000 P3 kit (Illumina).

### SS2

Single-cell complementary DNA was amplified from the 96-well plates containing the captured mRNA according to the SS2 protocol^27^ with slight modification for the nucleus.

In brief, oligo(dT)-primed reverse transcription was performed using Maxima H Minus reverse transcriptase (Thermo, EP0753) and locked TSO oligonucleotide (Supplementary Table 5). This was followed by PCR amplification (22 cycles for snRNA-seq) using KAPA HiFi HotStart ReadyMix (KAPA Biosystems, K2602) with subsequent Agencourt AMPure XP bead (Beckman, A63882) purification. Libraries were tagmented using the Nextera XT Library Prep kit (Illumina, FC-131-1096) with custom barcode adaptors. Libraries from 768 cells with unique barcodes were pooled and sequenced on NextSeq 2000 (Illumina).

### WES analysis

WES was performed as follows. DNA was extracted from each frozen tumor tissue and blood sample corresponding to the patient using the DNeasy Blood & Tissue kit (Qiagen, 69504). The gDNA (100–250 ng) was acoustically sheared by ultrasonicator (Covaris), targeting 150 bp fragments. Library preparation was performed by KAPA HyperPrep Kit (KAPA Biosystems, KK8504), followed by enzymatic clean-up using AMPure XP beads (Beckman, A63882). Exome capture was performed using a custom exome bait (manufactured by Twist Biosciences to the Broad Institute’s specifications). Captured libraries were sequenced with 150 bp paired-end sequencing on a NovaSeq 6000 (Illumina).

### Somatic variant detection and copy number calling

Single nucleotide variant and indel calls were done with Mutect2 in GATK-4.0.4.0. Detected mutations were annotated with Oncotato 1.9.8.0. Copy number variation calls were done with GATK-CNV in GATK-4.1.2.0. The workflow used a panel of normals to normalize coverage of input BAMs and removed artifacts originating from technical bias for estimating accurate CNAs. The CNA calls were annotated by Oncotator 1.9.5.0. Hypermutator was defined by segmented linear regression analysis as previously reported^52^.

### Analysis of TCGA patient samples

TCGA DNA methylation array data were downloaded from the TCGA database (https://cancergenome.nih.gov). The data for IDH-As classified as ‘G-CIMP-high’ or ‘G-CIMP-low’ in prior work^24^ and from IDH-Os classified as ‘codel’ were used in this study. G-CIMP score for TCGA methylation array data was calculated by averaging the beta values of all CpG probes within 1,000 bp windows around 684 CpG sites that showed significantly lower methylation values in G-CIMP-low tumors in a previous study^23^.

### Survival analysis

Survival analysis was performed using the R packages survival and survminer. Kaplan–Meyer estimates were computed using the survfit function and plotted using the ggsurvplot function (survminer package). Statistical significance of the difference between the survival curves was computed using the log-rank test.

### snRNA-seq analysis (3′ 10x)

Data were processed using Cell Ranger (v.6.0.0) using the mkfastq function and the count function (--include-introns --chemistry=threeprime arguments). The genome and gene annotation were obtained from 10x Genomics (v.GRCh38-2020-A). We included nuclei with more than 750 genes captured and more than 1,000 reads, retaining a total of 158,143 nuclei (mean, 4,350.5; range, 1,309–8,381) across samples that passed quality control with a mean ± s.e.m. of 3,125 ± 3 genes and 8,017 ± 15 reads captured per nucleus. Clustering was performed using the standard workflow from Seurat (v.4) using the top 2,000 variable genes and the top ten principal components for uniform manifold approximation and projection (UMAP) visualization. Doublets were removed based on the DoubletFinder R package. Cell types were labeled based on the correlation of gene expression with mean cell-type expression profiles from scRNA-seq data in the Allen Institute Brain Atlas (https://portal.brain-map.org/atlases-and-data/rnaseq - Human brain M1 - 10x Genomics 2020 and Smart-seq 2019). Malignant cells were classified based on the presence of CNAs called using the inferCNV R package (non-malignant cells identified as myeloid and oligodendrocytes were used as reference cells). Malignant cells were further classified as stem-like, OC-like or AC-like based on previously described methods^19,20^.

### snRNA-seq analysis (SS2)

Sequenced read fragments were mapped against the GRCh38 genome assembly (10x) using the 2pass default mode of STAR100 (v.2.5.2a). We included nuclei with more than 1,000 genes captured and more than 1 × 10^5^ reads. These retained a mean of 58.8 (range, 24–85) nuclei per sample with a mean ± s.e.m. of 2,987 ± 27 genes and 889,254 ± 6,624 reads per nucleus (total *n* = 2,117). Cell types were labeled based on the correlation of gene expression with mean cell-type expression profiles from scRNA-seq data in the Allen Institute Brain Atlas (https://portal.brain-map.org/atlases-and-data/rnaseq - Human brain M1 - 10x Genomics 2020 and Smart-seq 2019). Malignant cells were classified based on the presence of CNAs called using the inferCNV R package (non-malignant cells identified as myeloid and oligodendrocytes were used as reference cells). Malignant cells were further classified as stem-like, OC-like or AC-like based on previously described methods^17,19^.

### snXRBS analysis

Analysis was performed as described in a previous study^26^. Each pool of 96 cells was first demultiplexed by Illumina i7 barcodes, resulting in four pools of 24 cells. Each pool of 24 cells was further demultiplexed by unique cell barcodes. Quality control, trimming and alignment were then performed using bismark (v.0.22.1); mapping was performed using bowtie2 (v.2.2.8). Cells with coverage of at least 40,000 unique CpGs were retained for downstream analysis. This resulted in 2,117 (matched with nuclei that pass quality control for RNA) with a mean ± s.e.m. of 2,532,994 ± 17,548.85 total reads and 371,353 ± 3,405.415 CpG sites per nucleus. To score each cell based on G-CIMP status (G-CIM score), we used the 684 CpG previously defined as hypomethylated in recurrent glioma tumors compared to primary tumors. To account for sparsity in the single-nuclei data, we took the mean methylation of all CpG sites in a 1 kb window around each CpG site. We used the mean methylation in 100 kb bins across the genome to cluster nuclei based on DNA methylation profiles. Missing values for bins with no coverage were imputed using knn (R package), and principal component analysis was performed on the imputed values. To correct for sample-specific clustering, we used Harmony based on the first 15 principal components and generated a UMAP cell embedding using the umap function (v.0.2.3.1) with default settings. Cell type assignment based on RNA data was overlaid onto the UMAP from DNA methylation profiles. To identify the genomic regions with CNA, we used the number of CpG sites captured in 2 Mb bins across the genome. The CNA score per cell per bin was calculated as the mean of the log_2_ ratio of the counts in the bin compared with all non-malignant cells for that bin. A heatmap for visualization of this score was generated to visualize the CNA scores across the genome for all cells. CNA sub-clones were detected in samples with more than two sub-clones based on the heatmap using hierarchical clustering and cutting the tree into groups (cutree function in R; Fig. 5b); sub-clone labels were used to calculate the heritability score using PATH^48^. To identify differentially methylated regions, we compared the mean methylation in 1 kb bins across the genome between G-CIMP-high (G-CIMP score of >0.5) and G-CIMP-low cells (G-CIMP score of <0.4). As previously described^26^, we used a generalized linear model to predict the DNA methylation for 1 kb between G-CIMP-high and G-CIMP-low cells and retained bins that were captured in X number of cells. We also accounted for patient variation as a covariate. We defined regions with a Student’s *t*-test *P* value of <0.05 (after Benjamini–Hochberg correction) and an absolute DNA methylation difference of ≥5% as differentially methylated. To address the overlap of differentially methylated region results with genomic features, we calculated their overlap with chromHMM annotation^37^ and gene annotations using the genomicRanges R package, based on an overlap of 500 bps with each 1 kb bin. DNA methylation-based lineage trees were generated by applying a tree-searching maximum-likelihood algorithm based on binary DNA methylation values as previously described^26^. The MPI version of IQ-TREE (v.1.6.9) was used with a substitution model based on the binary alignment to infer a maximum-likelihood tree and compute bootstrap support values (1,000 bootstrap replicates). We opted for the automatic model selection procedure (-m TESTNEW), which implements the FreeRate heterogeneity model, inferring the site rates directly from the data instead of being drawn from a gamma distribution.

### Heritability and cell state transition analysis

As cell state heritability and plasticity are related to cell state variation, we measured transition dynamics using phylogenies that contained at least ten cells in each of the stem and differentiated cell states. Furthermore, we input scRNA-seq-based measurements of cell cycling to infer cell state transitions and removed samples without matched cell cycle scoring. To infer cell state transitions, we applied PATHpro, setting alpha = 0 and input estimated cell state proliferation rates from matched cell cycle scoring. To account for our phylogenetic branch lengths not measuring time, we input a range of estimated time-scaled branch lengths, rescaling phylogenetic branch lengths by a factor of ‘tscale’, and input t = mean.pat/tscale into PATHpro. Each PATHpro search per tree was run 256 times and replicated for each tscale, which ranged between 1 and 10^−3^, using intervals of 0.05. We then calculated Spearman’s correlation between both differentiation (exp *Q*_12_) and de-differentiation (exp *Q*_21_) transition probabilities with sample G-CIMP status and plotted these against each branch time scaling (Extended Data Fig. 8b). To determine how transitions changed between primary and recurrent samples in the same patient, we calculated the difference in stemness heritability (Extended Data Fig. 8a) and cell state transitions (recurrent minus primary, using tscale = 0.1; Fig. 5a). For Fig. 5b, heritability was calculated for a subset of tumors based on variance of the different features as follows: (1) G-CIMP heritability, tumors with variance of G-CIMP score greater than the median across all tumors; (2) stemness heritability was calculated for tumors with at least ten stem-like cells; (3) CNA sub-clone heritability was calculated for tumors with at least two detectable sub-clones based on hierarchical clustering; and (4) total reads heritability (negative control) was calculated for all tumors.

### Reporting summary

Further information on research design is available in the Nature Portfolio Reporting Summary linked to this article.

## Online content

Any methods, additional references, Nature Portfolio reporting summaries, source data, extended data, supplementary information, acknowledgements, peer review information; details of author contributions and competing interests; and statements of data and code availability are available at 10.1038/s41588-026-02642-7.

## Supplementary information

Reporting Summary

Supplementary Table Supplementary Table 1. Cohort and dataset. Supplementary Table 2. snXRBS statistics, G-CIMP score and cell state module score based on snRNA-seq for cell. Supplementary Table 3. 1 kb DMR malignant. Supplementary Table 4. Differential gene expression analysis between G-CIMP high and low tumors based on two methods. Supplementary Table 5. Primer sequences used in this study

## Data Availability

Processed data generated for this study are available at the Gene Expression Omnibus: GSE292025 (snXRBS), GSE291885(snRNA-seq; SS2), GSE292130 (snRNA-seq; 10x Genomics). In accordance with consent forms, raw sequencing data are deposited in an access-restricted database and are available upon request from the Data Use Oversight System (DUOS) at https://duos.broadinstitute.org under IDs DUOS-000475, DUOS-000476 and DUOS-000480. Researchers need to submit a single request form to request access to the datasets. The corresponding data access committees review these requests and provide access to approved researchers within a few weeks.

## Code availability

Analysis scripts used in this study are available at GitHub (https://github.com/rr1859/sc_DNAme_RNA_GCIMP_Analysis) and Zenodo (10.5281/zenodo.20097728)^53^.
