# Supplementary material for: Longitudinal changes in DNA methylation in IDH-mutant glioma fuel disease progression through altered cell state differentiation
Source: Nat Genet. 2026 Jun 22;58(7):1651–60. doi: 10.1038/s41588-026-02642-7 (PMC13364636; doi:10.1038/s41588-026-02642-7)
Supplement: Supplementary file 1 — Reporting Summary [file 41588_2026_2642_MOESM1_ESM.pdf]

nature portfolio

Corresponding author(s): Mario L. Suva, Dan A. Landau

Last updated by author(s): May 1, 2026

Reporting Summary

Nature Portfolio wishes to improve the reproducibility of the work that we publish. This form provides structure for consistency and transparency in reporting. For further information on Nature Portfolio policies, see our Editorial Policies and the Editorial Policy Checklist.

Statistics

For all statistical analyses, confirm that the following items are present in the figure legend, table legend, main text, or Methods section.

n/a	Confirmed	
☐	☒	The exact sample size (*n*) for each experimental group/condition, given as a discrete number and unit of measurement
☐	☒	A statement on whether measurements were taken from distinct samples or whether the same sample was measured repeatedly
☐	☒	The statistical test(s) used AND whether they are one- or two-sided<br>*Only common tests should be described solely by name; describe more complex techniques in the Methods section.*
☐	☒	A description of all covariates tested
☐	☒	A description of any assumptions or corrections, such as tests of normality and adjustment for multiple comparisons
☐	☒	A full description of the statistical parameters including central tendency (e.g. means) or other basic estimates (e.g. regression coefficient) AND variation (e.g. standard deviation) or associated estimates of uncertainty (e.g. confidence intervals)
☐	☒	For null hypothesis testing, the test statistic (e.g. *F*, *t*, *r*) with confidence intervals, effect sizes, degrees of freedom and *P* value noted<br>*Give P values as exact values whenever suitable.*
☒	☐	For Bayesian analysis, information on the choice of priors and Markov chain Monte Carlo settings
☒	☐	For hierarchical and complex designs, identification of the appropriate level for tests and full reporting of outcomes
☐	☒	Estimates of effect sizes (e.g. Cohen's *d*, Pearson's *r*), indicating how they were calculated

Our web collection on statistics for biologists contains articles on many of the points above.

Software and code

Policy information about availability of computer code

Data collection	No software was used for data collection.
Data analysis	Data analysis was performed in R v4.1.1. Packages used and versions follow below.<br>survminer_0.4.9, survival_3.3-1, viridis_0.6.2, viridisLite_0.4.0, RColorBrewer_1.1-3, NMF_0.24.0, synchronicity_1.3.5, bigmemory_4.5.36, Biobase_2.52.0, BiocGenerics_0.38.0, cluster_2.1.3, rngtools_1.5.2, pkgmaker_0.32.2, registry_0.5-1, igraph_1.3.0, BiocParallel_1.26.2, purrr_1.0.1, Matrix_1.3-4, circlize_0.4.13, ComplexHeatmap_2.8.0, scalop_1.1.0, infercna_1.0.0, ggridges_0.5.3, ggpubr_0.4.0, reshape2_1.4.4, scales_1.2.1, ggplot2_3.4.1, patchwork_1.1.1, SeuratObject_4.0.2, Seurat_4.0.4, tibble_3.1.6, dplyr_1.1.0.<br>Analysis scripts used in this study is available at Github (https://github.com/rr1859/sc_DNAme_RNA_GCIMP_Analysis).

For manuscripts utilizing custom algorithms or software that are central to the research but not yet described in published literature, software must be made available to editors and reviewers. We strongly encourage code deposition in a community repository (e.g. GitHub). See the Nature Portfolio guidelines for submitting code & software for further information.

Data

Policy information about availability of data

All manuscripts must include a data availability statement. This statement should provide the following information, where applicable:

- Accession codes, unique identifiers, or web links for publicly available datasets
- A description of any restrictions on data availability
- For clinical datasets or third party data, please ensure that the statement adheres to our policy

Processed data generated for this study will be available at the Gene Expression Omnibus. GSE292025 (snXRBS), GSE291885 (snRNA-seq, Smart-seq2), GSE292130 (snRNA-seq, 10X genomics). Raw sequencing data is available with limitations in accordance with the consent forms from the Data Use Oversight System (DUOS) at https://duos.broadinstitute.org. under IDs: DUOS-000475; DUOS-000476; and DUOS-000480. The Cancer Genome Atlas (TCGA) data is available under TCGA portal (https://cancergenome.nih.gov/). single-cell multi-omics dataset from a previous study (Chaligne et al., Nat Genet., 2021) is available in GSE151506.

Research involving human participants, their data, or biological material

Policy information about studies with human participants or human data. See also policy information about sex, gender (identity/presentation), and sexual orientation and race, ethnicity and racism.

Reporting on sex and gender	The study analyzed IDH mutant glioma. Patients were male and female. The covariate-relevant population characteristics of the human research participants are provided in Supplementary Table 1.
Reporting on race, ethnicity, or other socially relevant groupings	IDH mutant glioma samples longitudinally collected in 3 hospitals were analyzed in this study.
Population characteristics	Human IDH mutant gioma samples were provided by 3 hospitals in 3 countries (USA, Canada and Japan). The covariate-relevant population characteristics of the human research participants are provided in Supplementary Table 1.
Recruitment	This is a retrospective study of archival specimens, for which written informed consent was obtained. All viable primary and matched recurrent IDH mutant frozen samples that were longitudinally collected in 3 hospitals were used in this study.
Ethics oversight	Collection was approved by the Institutional Review Board of following institutions, and all patients provided informed consent accordingly: The Institutional Review Board of MD Anderson Cancer Center (protocol number 2012-0441), the Institutional Review Board of Tokyo University Hospital (protocol number G10028) and the Institutional Review Board of St. Michael's Hospital (protocol number REB # 13-14).

Note that full information on the approval of the study protocol must also be provided in the manuscript.

Field-specific reporting

Please select the one below that is the best fit for your research. If you are not sure, read the appropriate sections before making your selection.

☒ Life sciences ☐ Behavioural & social sciences ☐ Ecological, evolutionary & environmental sciences

For a reference copy of the document with all sections, see nature.com/documents/nr-reporting-summary-flat.pdf

Life sciences study design

All studies must disclose on these points even when the disclosure is negative.

Sample size	Sample size was determined by the availability of donor and patient-derived material. We performed single-nucleus sequencing for 36 samples.
Data exclusions	No data were excluded from the study.
Replication	This study doesn't include in vivo or in vitro experiments. We performed replicates for snRNA-seq by generating 10X and Smart-seq2 data. Part of findings of single-nucleus XRBS analysis was validated by public bulk DNA methylation array analyses.
Randomization	Randomization is not applicable as no experimental groups were used in this study.
Blinding	Blinding was not applicable to this study since no effect for treatment or perturbations to the system were assesed.

Reporting for specific materials, systems and methods

We require information from authors about some types of materials, experimental systems and methods used in many studies. Here, indicate whether each material, system or method listed is relevant to your study. If you are not sure if a list item applies to your research, read the appropriate section before selecting a response.

Materials & experimental systems

n/a	Involved in the study
☒	☐ Antibodies
☒	☐ Eukaryotic cell lines
☒	☐ Palaeontology and archaeology
☒	☐ Animals and other organisms
☒	☐ Clinical data
☒	☐ Dual use research of concern
☒	☐ Plants

Methods

n/a	Involved in the study
☒	☐ ChIP-seq
☐	☒ Flow cytometry
☒	☐ MRI-based neuroimaging

Plants

Seed stocks	*Report on the source of all seed stocks or other plant material used. If applicable, state the seed stock centre and catalogue number. If plant specimens were collected from the field, describe the collection location, date and sampling procedures.*
Novel plant genotypes	*Describe the methods by which all novel plant genotypes were produced. This includes those generated by transgenic approaches, gene editing, chemical/radiation-based mutagenesis and hybridization. For transgenic lines, describe the transformation method, the number of independent lines analyzed and the generation upon which experiments were performed. For gene-edited lines, describe the editor used, the endogenous sequence targeted for editing, the targeting guide RNA sequence (if applicable) and how the editor was applied.*
Authentication	*Describe any authentication procedures for each seed stock used or novel genotype generated. Describe any experiments used to assess the effect of a mutation and, where applicable, how potential secondary effects (e.g. second site T-DNA insertions, mosiacism, off-target gene editing) were examined.*

Flow Cytometry

Plots

Confirm that:

☒ The axis labels state the marker and fluorochrome used (e.g. CD4-FITC).

☒ The axis scales are clearly visible. Include numbers along axes only for bottom left plot of group (a 'group' is an analysis of identical markers).

☒ All plots are contour plots with outliers or pseudocolor plots.

☒ A numerical value for number of cells or percentage (with statistics) is provided.

Methodology

Sample preparation	Nuclei from frozen tumor tissue were isolated as previously reported52. Briefly, tumor tissue was thawed and mechanically dissociated in ST buffer (10mM Tris-HCL pH7.5, 1mM CaCl2, 146mM NaCl, 21mM MgCl2) with 0.49% CHAPS (Millipore, 28300). Single-nuclei suspensions were filtered using a 40 µm strainer, centrifuged at 500g for 5min, and resuspended in ST buffer supplemented with 0.01% bovine serum albumin (NEB, B9000S). Nucleus suspensions were inspected by microscope, counted using a hemocytometer, and used for FACS-sorting for single-nucleus dual sequencing workflow.
Instrument	Aria Fusion sorter (Becton Dickinson)
Software	FACSDiva software v8.0.1
Cell population abundance	For nuclei dissociation from frozen tumors, the Vybrant Ruby Stain positive event rate ranged from 10-90%.
Gating strategy	Single nuclei were identified by positive staining for Vybrant Ruby Stain and singlet gating (laser light area vs height).

☒ Tick this box to confirm that a figure exemplifying the gating strategy is provided in the Supplementary Information.
